# *Mycobacterium ulcerans* DNA in Bandicoot Excreta in Buruli Ulcer–Endemic Area, Northern Queensland, Australia

**DOI:** 10.3201/eid2312.170780

**Published:** 2017-12

**Authors:** Katharina Röltgen, Gerd Pluschke, Paul D.R. Johnson, Janet Fyfe

**Affiliations:** Swiss Tropical and Public Health Institute, Basel, Switzerland (K. Röltgen, G. Pluschke);; University of Basel, Basel (K. Röltgen, G. Pluschke);; Austin Health, Heidelberg, Victoria, Australia (P.D.R. Johnson);; University of Melbourne, Parkville, Victoria, Australia (P.D.R. Johnson);; World Health Organization Collaborating Centre for *Mycobacterium ulcerans,* Melbourne, Victoria, Australia (P.D.R. Johnson, J. Fyfe);; Doherty Institute, Melbourne (J. Fyfe)

**Keywords:** Buruli ulcer, animal reservoir, Mycobacterium ulcerans, bacteria, DNA, insertion sequence element IS2404, tuberculosis and other mycobacteria, mycolactone-producing mycobacteria, bandicoot, excreta, zoonoses, Far North Queensland, Queensland, Australia, Switzerland

## Abstract

To identify potential reservoirs/vectors of *Mycobacterium ulcerans* in northern Queensland, Australia, we analyzed environmental samples collected from the Daintree River catchment area, to which Buruli ulcer is endemic, and adjacent coastal lowlands by species-specific PCR. We detected *M. ulcerans* DNA in soil, mosquitoes, and excreta of bandicoots, which are small terrestrial marsupials.

*Mycobacterium ulcerans* infections, which cause the chronic, necrotizing skin disease Buruli ulcer (BU), occur mainly in focalized areas in West Africa but have also been reported in Australia, Asia, and the Americas (*1*). Much of the pathology of this debilitating disease is caused by mycolactone, a macrolide toxin (*2*) unique for members of the species *M. ulcerans*, which are also referred to as mycolactone-producing mycobacteria (*3*).

Although the definite route of infection with *M. ulcerans* remains obscure, in Victoria, Australia, small arboreal marsupials (possums) have been implicated as reservoirs of the pathogen, and mosquitoes have been implicated as vectors of the pathogen (*4**,**5*). In a second BU-endemic area of Australia, in Far North Queensland (*6*), a similar animal reservoir has not been identified. In this region, outbreaks of BU occur in waves, separated by several years, and are believed to be associated with environmental changes caused by heavy rainfall.

The major region in Far North Queensland to which BU is endemic is a rim of valleys and lowlands surrounding the Dagmar Range and extending from Daintree and Forest Creek in the northern region to Mossman in the southern region (*6*). In this area, a BU outbreak with 64 reported cases occurred in 2011 after the exceptionally long and wet rainy season during 2010–2011, which led to flooding of the Daintree River basin (*7*). An additional 23 cases were identified in 2012, but numbers subsequently reported have been sporadic.

Although proximity to stagnant water bodies and hydromorphologic alterations are well-established risk factors for emergence of *M. ulcerans* infection foci (*8*), definite ecologic factors leading to focal emergence of BU in humans have not been determined. The aim of this study was to identify potential reservoirs and vectors of *M. ulcerans* in the BU-endemic area of Far North Queensland by analyzing environmental samples for the presence of species-specific DNA sequences.

## The Study

**Figure 1:**
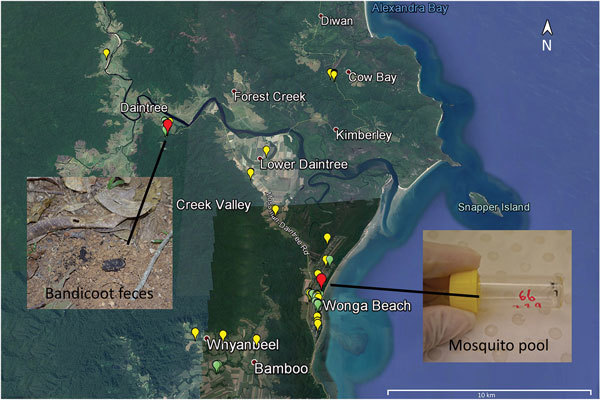
Sample collection for detection of *Mycobacterium ulcerans* DNA in Buruli ulcer–endemic area, northern Queensland, Australia. Environmental samples were collected in the Daintree River basin during September (yellow) and October (green) 2013. Red indicates locations where Bandicoot feces and mosquito pool samples with positive results by real-time PCR for all 3 *M. ulcerans* targets were collected. Inset shows specimens from bandicoots and mosquitoes. Map created by using Google Earth (https://www.google.com/earth/).

We collected 102 environmental samples (55 from soil/mud/vegetation, 35 from insects or small insect pools, and 12 from animal excreta) in September 2013 from different locations within the Daintree River basin, in which BU cases were reported during the outbreak in 2011. Global positioning system coordinates were recorded for all sampling locations (Figure 1). Specimens were collected in sterile plastic containers and shipped to the Victorian Infectious Disease Reference Laboratory (Melbourne, Victoria, Australia) for PCR analysis.

We extracted DNA by using the FastPrep Instrument (MP Biomedicals, Solon, OH, USA) as described (*4*). We used the FastDNA Kit (MP Biomedicals) for insect samples and the FastDNA Spin Kit (MP Biomedicals) for soil, mud, vegetation, and feces samples. We analyzed DNA extracts by using semiquantitative real-time PCRs optimized for detection of *M. ulcerans* in environmental samples (*9*). We first screened all extracted DNA samples for *M. ulcerans* insertion sequence element IS*2404*. Subsequently, we analyzed IS*2404*-positive samples in a second real-time PCR to detect 2 additional regions in the genome of *M. ulcerans*: IS*2606* and a sequence encoding the ketoreductase B domain of the mycolactone polyketide synthase genes.

**Figure 2:**
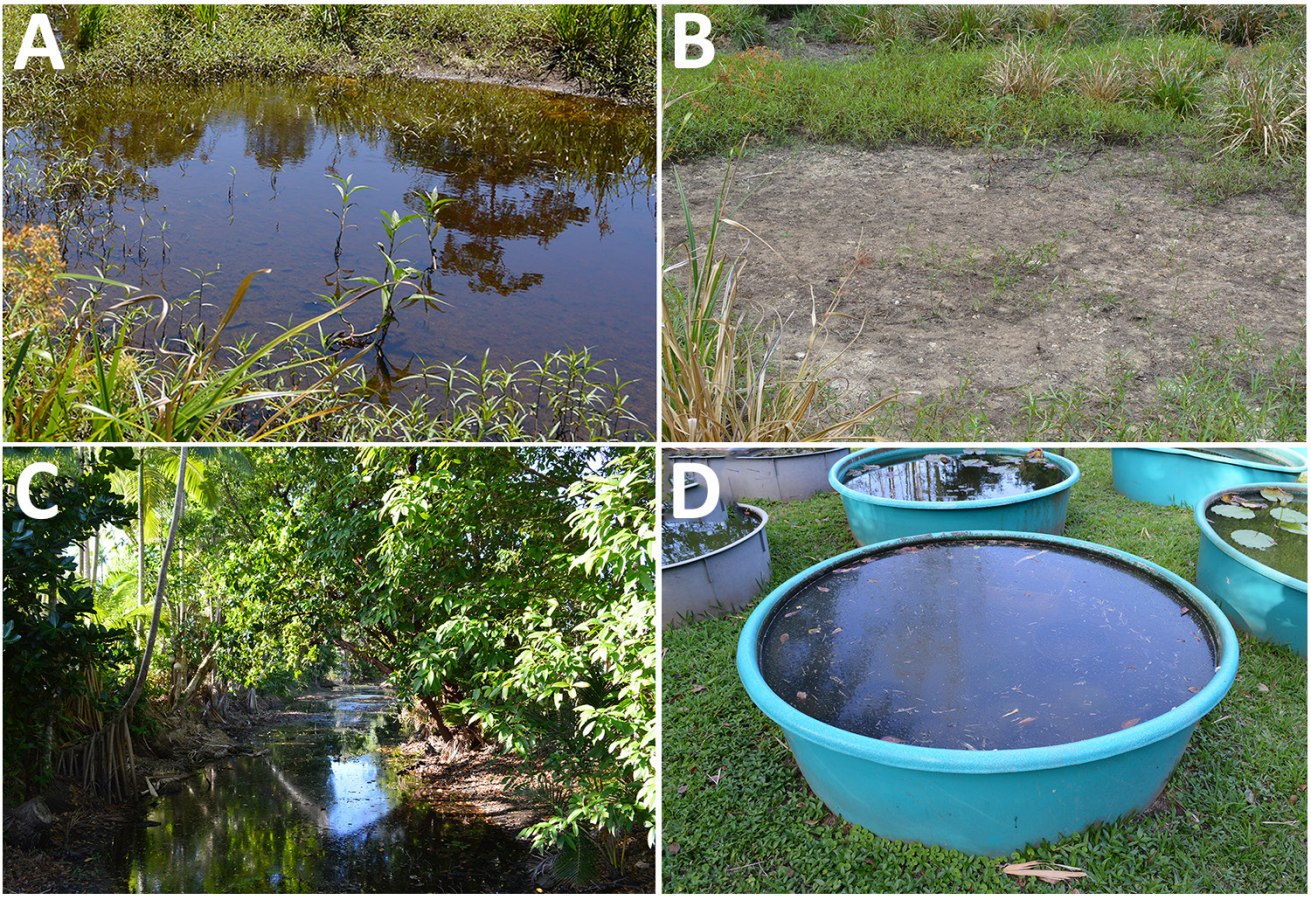
Sample locations for detection of *Mycobacterium ulcerans* DNA in Buruli ulcer–endemic area, northern Queensland, Australia. A) Pond where IS*2404*-positive soil sample was collected at first sampling time in September 2013; B) same location dried out at the end of the dry season in October. C, D) Other water bodies suspected to be linked to *M. ulcerans* infections, such as creeks (C) or water surfaces near houses (D), showed negative results for IS*2404*. IS, insertion sequence.

Of the 102 samples, 5 (1 soil specimen, 2 bandicoot [*Isoodon macrourus*] feces samples, 1 sample of an individual mosquito, and 1 pool of 2 mosquitoes**)** were positive for IS*2404*. Although 3 of the 5 specimens did not contain sufficient amounts of DNA to identify IS*2606* and the ketoreductase B domain of the mycolactone polyketide synthase genes, as indicated by the high cycle threshold (C_t_) values for multicopy IS*2404* (Table), these markers were detectable in the other 2 samples (bandicoot feces and the pool of 2 mosquitoes) (Figure 1). The IS*2404*–positive soil sample and the 2 bandicoot feces specimens were collected at the same location (Figure 1), close to a small pond (Figure 2, panel A), and 2 positive mosquito samples were collected at Wonga Beach (Figure 1).

**Table:** Molecular genetic analysis of environmental samples for *Mycobacterium ulcerans* DNA in Buruli ulcer–endemic area, northern Queensland, Australia*

Sample	Real-time PCR analysis			
	IS*2404*	IS*2606*	IS*2606*–IS*2404*	KR
Bandicoot feces 1†	37.3	ND	ND	ND
Bandicoot feces 2†	27.8	29.1	1.3	28.9
Soil	36.3	ND	ND	ND
Mosquito	39.2	ND	ND	ND
Mosquito pool	31.0	38.6	7.6	31.9
Bandicoot feces‡	31.4	33.9	2.5	33.9

*Values are mean cycle thresholds of duplicate tests. IS, insertion sequence; KR, ketoreductase B domain of mycolactone polyketide synthase genes; ND, not detected. †Collected in September 2013. ‡Collected in October 2013.

As reported (*9*), analysis of difference in real-time PCR cycle thresholds between IS*2606* and IS*2404* (ΔC_t_ [IS*2606* – IS*2404*]) enables differentiation between strains of *M. ulcerans* known to cause BU in humans and other mycolactone-producing mycobacteria strains that contain IS*2404* but have fewer copy numbers of IS*2606* and are not known to cause disease in endotherms. Thus, DNA in bandicoot feces could be attributed to the *M. ulcerans* genotype known to cause BU (Table). Conversely, the mosquito pool contained DNA of a closely related *M. ulcerans* subspecies that had a low copy number for IS*2606* (Table), which is usually not associated with disease in endotherms.

The location at which the bandicoot specimen containing *M. ulcerans* DNA was collected was situated in a nature refuge. In October 2013, we performed a followup environmental study that focused on environmental samples, predominantly animal excreta, collected in this refuge. Of 18 soil/mud/vegetation specimens, 12 insects/insect pools, and 74 animal excreta samples collected, only 1 sample, a bandicoot feces specimen found at almost the same location as the *M. ulcerans*–positive bandicoot feces specimens collected during the first sampling (Figure 1), was also positive for all *M. ulcerans* DNA markers tested (Table).

## Conclusions

Motivated by increased evidence for the role of *M. ulcerans*–infected possums in the ecology of human BU in southeastern Australia (*4**,**5*), we searched for a similar animal reservoir of *M. ulcerans* in a BU-endemic area of northern Queensland. Detection of *M. ulcerans* DNA in bandicoot feces at 2 sampling time points separated by 4 weeks suggests that small mammals might be a potential reservoir for this pathogen. Our data provide a basis for future investigations, which should include a survey of the local animal population for lesions of BU. Similar studies have not identified a mammalian animal reservoir in BU-endemic regions of West Africa (*10*). Therefore, investigations should be conducted to determine whether at least some marsupial species are more susceptible to *M. ulcerans* infection than other mammals.

In Far North Queensland, the Daintree River obtains its waters from the mountainous rainforest region northwest of the small town of Mossman and flows into the sea at Cape Tribulation. The wet season is November/December–April, and the dry season is May–October/November. We encountered different environmental conditions at the 2 sampling time points. In September, water bodies, including creeks, small lakes, and swamps, were filled with water (Figure 2, panel A). However, water levels were comparatively low at the end of the dry season in October; some water bodies had even dried up (Figure 2, panel B). Samples from other water bodies, such as creeks (Figure 2, panel C) or biotopes near houses (Figure 2, panel D) in which the presence of *M. ulcerans* was suspected, all showed negative results for IS*2404*.

Because outbreaks of BU in Far North Queensland were historically related to heavy rainfalls and flooding, future studies in this region should be performed during or shortly after the rainy season. Although mosquitoes seem to be involved in the ecology of BU in Victoria (*5**,**11**,**12*), the mosquito sample positive for *M. ulcerans* in this study did not have a bacterial genotype that is known to commonly cause disease in endotherms.
